# Clinical Epidemiology of Chickenpox in Iraq from 2007-2011

**DOI:** 10.5539/gjhs.v5n1p180

**Published:** 2012-11-22

**Authors:** Hanan Abdulghafoor Khaleel, Hassan Muslem Abdelhussien

**Affiliations:** 1 Viral Hepatitis Section, CDCC, Public Health Directorate, MOH, Iraq; 2 Public Health Directorate, MOH, Iraq

**Keywords:** chickenpox, varicella zoster, Iraq, prevalence, vaccine

## Abstract

Varicella zoster (chickenpox) infection is an acute common disease caused by the varicella zoster virus (VZV). Children are most susceptible to infection. In non-vaccinated populations, primary infection tends to occur at a younger age. In 1998, the World Health Organization (WHO) recommended that routine childhood varicella vaccination be considered in countries where the disease is a relatively important public health and socioeconomic problem, and where high (85 to 90%) and sustained vaccine coverage can be achieved.

**Aim:**

1- To determine the trend (occurrence, age and gender distribution, seasonal variation) of registered clinical cases of chickenpox in Iraq from 2007-2011.2- To determine the need for the use of chicken pox vaccine in Iraq and putting a plan for its use accordingly.

**Methods:**

Retrospective descriptive study.

**Results:**

Frequency of clinical cases shows an obvious rise in the registration of chickenpox cases from 21798 case in 2007 through 59681 in 2008 to 74195 in 2011 with possible outbreaks occurred in 2008 and 2011. Rate of occurrence of clinical chickenpox cases also shows an obvious rise in the occurrence that ranges from 73.41/100000 in 2007 to 222.61/100000 in 2011. The rate in 2008 and 2011 is suggestive of a possible outbreak. Although the total number of chickenpox varies from 2007-2011 but all have shown the same seasonal distribution, being highest in spring (April, May) season. The largest no. recorded was in 2011 (14000 cases in April and May). The lowest no. recorded was in 2007 (4000 cases in April and May).The highest registered number of chickenpox cases was in provinces of Ninawa, Baghdad/russafa, Dihok, Baghdad/karkh, Al-Basrah, As-Sulaymaniyah. Regarding gender distribution there was sustained preponderance for the males over females with nearly the same percentage over the years. Age distribution of the registered cases had shown that most of the cases occurred in those of age 5-14 years (65%), only 1% occur in those >45 years with statistical significance p=0.001.

**Conclusions:**

1- There is a rising trend in the registration of clinical chickenpox cases.2- Most cases occur in the age group of less than 15 years. Males are a little bit higher than females.3- The highest frequencies were reported in March, April, and May.4- Most of the cases were registered in Baghdad, Ninawa, Dihok and Al-Basrah.

## 1. Background

Varicella zoster (chickenpox) infection is an acute common disease caused by the varicella zoster virus (VZV) (Almuneef, 2006). Children are most susceptible to infection. In non-vaccinated populations, primary infection tends to occur at a younger age (Gershon, 2008). The disease can be benign and self-limiting in children, but in adults and immunocompromised hosts, it can be severe with high morbidity and mortality (Gregorakos, 2002). The occurrence of chicken pox is different in different geographical zones. In temperate countries chickenpox is usually a mild, self-limiting infection, affecting pre-school children (Vyse, 2004), however, the incidence of chickenpox in these areas is increasing in adolescents and adults (Fairley & Miller, 1996), which may in part be due to increased world travel and economic migration of susceptible individuals. In many tropical countries the epidemiology is different, with less than 60% of adults being immune (Lee, 1998). According to Aristotle’s classification of the earth’s climatic zones, Iraq is considered to be located in the temperate zones (Aristotle). For every 100,000 individuals who develop chickenpox, between four and nine will die, of whom 81-85% will be adults (Rawson et al., 2001; Brisson, 2003). Chickenpox is five times more likely to be fatal in pregnancy than in the non-pregnant adult (Department of Health, 2004). Although varicella is usually a benign childhood disease, and rarely rated as an important public health problem, varicella zoster virus may induce pneumonia or encephalitis, sometimes with persistent sequelae or death. Secondary bacterial infections of the vesicles may leave disfiguring scars or result in necrotizing fasciitis or septicaemia (Heymann, 2008). In 1998, the World Health Organization (WHO) recommended that routine childhood varicella vaccination be considered in countries where the disease is a relatively important public health and socioeconomic problem, where the vaccine is affordable, and where high (85 to 90%) and sustained vaccine coverage can be achieved (Paolo Bonanni, 2009). Universal vaccination programmes may cause an increase in the average age of infection, which may lead to increased adult morbidity and incidence of congenital varicella syndrome (CVS) and severe neonatal varicella (de Moira, 2005).

Chickenpox is considered as a monthly notifiable disease in Iraq (Respiratory infections section, 2012). The diagnosis is clinical. Clinical cases attending the primary health care centers all over the country are reported to the surveillance section in communicable diseases control center. Management is usually supportive. Data regarding hospitalization and death are lacking.

## 2. Aim

1 - To determine the trend (occurrence, age and gender distribution, seasonal variation) of registered clinical cases of chickenpox in Iraq from 2007-2011.
2 - To determine the need for the use of chicken pox vaccine in Iraq and putting a plan for its use accordingly.

## 3. Methods

A retrospective descriptive study was done. Review of the existing anonymous surveillance records of chicken pox for the years from 2007-2011 was done. Those monthly records were sent from corresponding surveillance units in Baghdad and other provinces which collect the data from primary health care centers. Excel 2010 was used for statistical analysis. Frequency and relative frequency were displayed; Bar graphs and line chart were used to present frequency distribution and seasonal variation. Chi square test (X^2^) was used to calculate the significance of the different frequency among gender and age groups from 2007-2011, each separately. P value was considered significant when it is less than 0.5. EpiInfo 3.5 was used to make the map of geographical distribution of cases. Age classification used in this study was based on age classification used in the surveillance system in Iraq.

Case definition used in primary health care centers to diagnose cases of chickenpox is:

Clinical illness that is characterized by a rash with rapid evolution of macules to papules, vesicles and crusts; all stages are simultaneously present; lesions are superficial and may appear in crops.

## 4. Results

**Figure 1:**
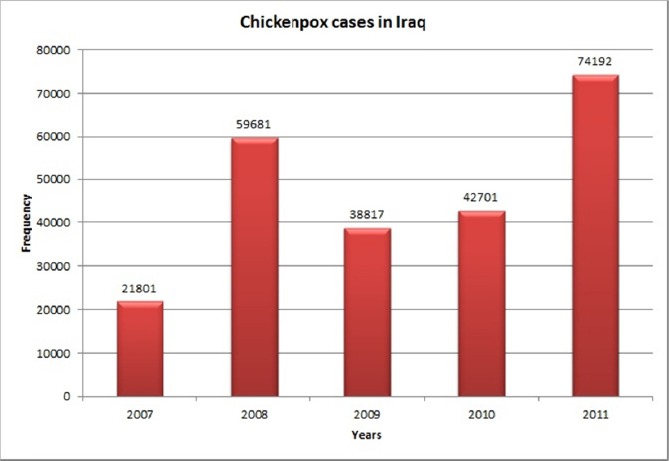
Frequency of clinically diagnosed chickenpox in Iraq from 2007-2011

**Figure 2:**
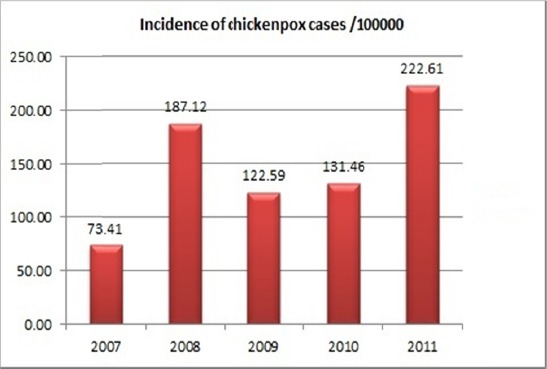
Rate of occurrence of clinical chickenpox cases in Iraq from 2007-2011

**Figure 3:**
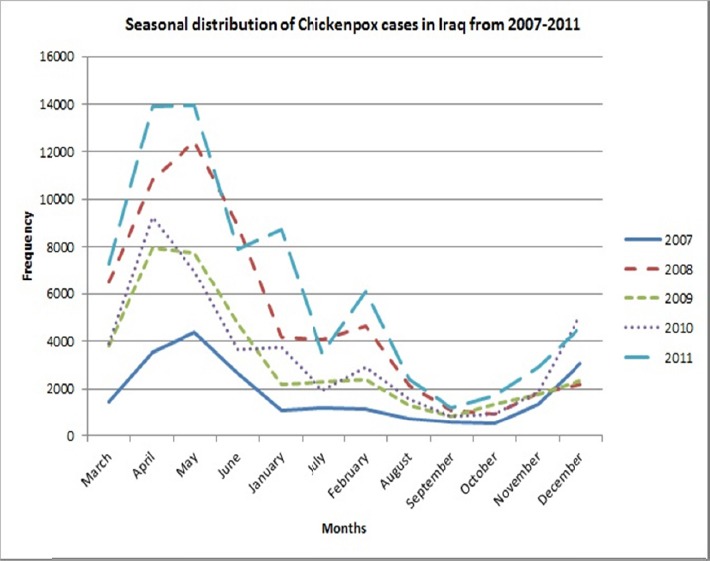
Seasonal distribution of clinically diagnosed chickenpox in Iraq from 2007-2011

Frequency of clinical cases shows an obvious rise in the registration of chickenpox cases from (21798 case) in 2007 through (59681) in 2008 to (74195) in 2011 with possible outbreaks (Respiratory infections section, 2012) (Suveillance Section, 2012) occurred in 2008 and 2011 (Figure 1). Rate of occurrence of clinical chickenpox cases also shows an obvious rise in the occurrence that ranges from 73.41/100000 in 2007 to 222.61/100000 in 2011. The rate in 2008 and 2011 is suggestive of a possible outbreak (Figure 2). Although the total number of chickenpox varies from 2007-2011 but all have shown the same seasonal distribution, being highest in spring (April, May) season. It shows that the start of rising cases is in December and January. The largest no. recorded was in 2011 (14000 cases in April and May). The lowest no. recorded was in 2007 (4000 cases in April and May) (Figure 3).

**Figure 4:**
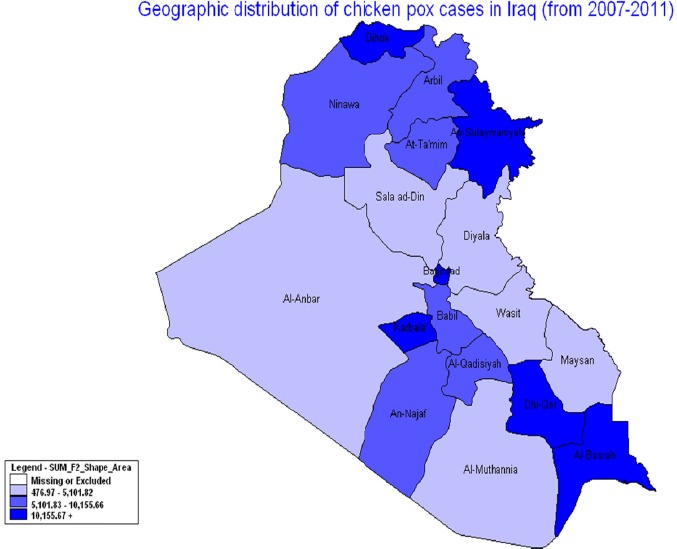
Geographic distribution of chickenpox cases in Iraq from 2007-2011

The highest registered number of chickenpox cases was in provinces of Ninawa (nearly 26000), Baghdad/russafa (245000), Dihok (24000), Baghdad/karkh (21000), Al-Basrah (nearly 20000), As-Sulaimanya (19000) (Figure 4). Regarding gender distribution there was sustained preponderance for the males over females with nearly the same percentage over the years (Table 1).

**Table 1:** Gender distribution of clinically diagnosed chickenpox in Iraq from 2007-2011

Year/Gender	Male		Female		Total

	N	%	N	%	
2007	11716	53.75	10082	46.25	21798
2008	31888	53.43	27793	46.57	59681
2009	21401	55.13	17416	44.87	38817
2010	23416	54.84	19285	45.16	42701
2011	40767	54.95	33428	45.05	74195

P=0.001

Age distribution of the registered cases had shown that most of the cases occurred in those of age 5-14 years (65%), only 1% occur in those >45 years with statistical significance p=0.001 (Table 2).

**Table 2:** Age distribution of clinical chickenpox cases in Iraq from 2007-2011

Year/Age group	<1 yr		1-4 yrs		5-14 yrs		15-44 yrs		>45 yrs		

	N	%	N	%	N	%	N	%	N	%	Total
2007	624	2.9	4199	19.3	14348	65.8	2422	11.1	205	0.9	21798
2008	1701	2.9	12820	21.5	38173	64.0	6463	10.8	524	0.9	59681
2009	1180	3.0	7530	19.4	25521	65.7	4057	10.5	529	1.4	38817
2010	1274	3.0	7512	17.6	28399	66.5	4575	10.7	941	2.2	42701
2011	2077	2.8	13828	18.6	48494	65.4	9005	12.1	788	1.1	74192

P=0.001

## 5. Discussion

There was an obvious rise in the registration of chickenpox cases from (21798 case) in 2007 to (74195) in 2011, these can be explained by possible outbreaks (Suveillance Section, 2012) occurred in 2008 and 2011 (Figure 1). These frequencies are much higher than what was reported in Saudi Arabia (Almuneef, 2006). Compared with rate of occurrence of clinical chickenpox cases it has also showed an obvious rise in the occurrence that ranges from 73.41/100000 in 2007 to 222.61/100000 in 2011 (Figure 2). The rate in 2008 and 2011 is suggestive of a possible outbreak. However, this rise can be attributed to increasing the number of primary health care centers that eased the access of the population to medical care whenever they need it.

Population-wide occurrence of varicella cases in Turkey, which is still in the prevaccine era, is estimated to be 466-768 per 100,000 children (Dinleyici, 2012). In Italy, Varicella incidence in 0-14 year-olds was 6136.8/100,000 person-years in 2000 and 4004.8 in 2008 after introduction of vaccine (Pozza, 2011). In Taiwan, a study has estimated the highest incidence of chickenpox was to be 540 cases per 100,000 populations (Liu, 1998). Estimated incidence in Catalonia was 762.2 per 100,000 persons/year according to the discussion above (Muñoz, 2001).

The introduction of the vaccine, within the routine schedule or in high risk groups is being raised because it has shown that it had reduced both the occurrence of the disease and its complication in childhood and herpez zoster in adulthood actually.

The decision of vaccination is determined by many factors such as the epidemiological situation, the coverage rate, costs and benefits. When an individual is vaccinated, costs and benefits accrue both to that individual directly and to the society as a whole (Jingzhou et al., 2011). Although vaccines are available, there are only a few countries with an early-childhood vaccination program. Most countries mainly focus on vaccination of high-risk groups, such as susceptible healthcare workers (Rozenbaum, 2008). One of the main concerns with a routine early-childhood vaccination program is a potential (temporal) increase of the incidence of herpes zoster among elderly adults (Rozenbaum, 2008). Vaccination of adolescents and adults will protect at-risk individuals, but will not have a significant impact on the epidemiology of the disease on a population basis. On the other hand, extensive use as a routine vaccine in children will have a significant impact on the epidemiology of the disease. If sustained high coverage can be achieved, the disease may virtually disappear. If only partial coverage can be obtained, the epidemiology may shift, leading to an increase in the number of cases in older children and adults. Hence, routine childhood varicella immunization programmes should emphasize high, sustained coverage (World Health Organization, 1998).

Although the total number of chickenpox varies from 2007-2011 but all have shown the same seasonal distribution, being highest in spring (April, May) season (Figure 2). It shows that the start of rising cases is in December and January. The largest no. recorded was in 2011 (14000 cases in April and May). The lowest no. recorded was in 2007 (4000 cases in April and May). These finding are similar to was reported in Saudi Arabia (Almuneef, 2006) and India (Lee, 1998) and can be considered as the Iraqi trend of the disease.

Geographic distribution of clinical chickenpox cases in Iraq showed that the highest registered number of chickenpox cases was in provinces of Ninawa, Baghdad/russafa, Dihok, Baghdad/karkh, Al-Basrah, As-Sulaimanya. Provinces of the middle Euphrates had shown the lowest registered number of cases (Figure 4). This might be attributed to either the climate of the middle Euphrates area, as it is dry hot and that might have an effect on the survival and infectivity of the virus or to the registration of the cases. It has been found the transmission of the virus is reduced in the humid hot climates (Lee, 1998).

Gender distribution of the cases throughout the years has shown sustained preponderance for the males over females (Table 1). This might be attributed to the social preference and care for the males in the Iraqi community that leads to seeking health care for the ill male more than for ill female.

However, there were a number of limitations to this study. Firstly, there was lack of data regarding hospitalizations, complications and death from varicella in addition to lack of data regarding the occurrence of the disease in pregnant women, immunocompromized and infants. Furthermore, report according to age and gender has not started until 2007. Underreporting of cases may give fake epidemiological situation. Lastly, lack of laboratory confirmation of the clinical cases and no information of the serological changes of the individuals was also a limit to this study.

Chickenpox, a highly transmissible childhood disease that becomes more severe with age, has a 10–30 times greater mortality rate for adults than for juveniles (Lenne et al., 2006). This study shows that most of the cases occurred in those of age 5-14 years (65%), only 1% occur in those >45 years with statistical significance p=0.001 (Table 2).In temperate climates, at least 90% of the population has had varicella disease by the age of 15 years and at least 95% of the population by young adulthood (Alberta Health and Wellness, 2012). In tropical countries the epidemiology of the disease is quite different. In these areas a higher proportion of cases occur among adults (Alberta Health and Wellness, 2012).

From this study, it’s clearly obvious that there is a rising trend in the registration of clinical chickenpox cases. Furthermore, we can find that most cases occur in the age group of less than 15 years and males are a little bit higher than females. Regarding seasonal distribution, the highest frequencies were reported in March, April, and May. Finally, most of the cases were registered in Baghdad, Ninawa, Dihok and Al-Basrah. Based on what has been mentioned before, the following are recommended:

1 - A national survey for the prevalence of markers of infection with varicella zoster should be conducted. The aim of the survey is to determine the prevalence of seromarkers of infection with varicella zoster among the adults. This estimate may provide a clue about naturally immunized persons and those who needs to be vaccinated.
2 - Establishment of case based surveillance of chicken pox cases and report any (hospitalization, complications and deaths) caused by chicken pox in order to determine the burden of the disease in the country. The use of laboratory confirmation of cases is essential especially for the high risk groups.
3 - Surveillance of chickenpox cases among pregnant women and immunocompromized patients in order to plan management for chickenpox during pregnancy and the need for varicella immunoglobulin.
4 - Collaboration with the ACIP (American Council for Immunization Programme) to put a clear plan of introducing the chickenpox vaccine into the immunization program in Iraq after complete understanding of the real epidemiological situation of the disease in Iraq.

## Authors’ Contribution

Dr. Hanan Abdulghafoor Khaleel: Review of Literature, Tables and Figures, Discussion.

Dr. Hassan Muslem Abdelhussien: Methods, Results, Revision of the article.

The article had displayed the situation of chickenpox in Iraq from many aspects including the method used in diagnosis, the frequency of cases and the need for vaccine. Although the vaccine has a great effect in reducing the occurrence of the disease but its use is not an easy task. It should be done based on clear understanding of the epidemiological situation of the disease in the country in order to avoid age shift. This study, although have not covered all the epidemiological aspects but at least could be considered as a beginning for future studies to highlight the real situation.
